# The Impact of Psychosocial Aspects as Risk Factors for Long-Term Illnesses in General Practice: Protocol for a Scoping Review

**DOI:** 10.2196/102585

**Published:** 2026-10-21

**Authors:** Kathrin Epple, Julia Nothacker, Lisa Richter, Christin Löffler, Ralf Jendyk, Andreas Dierich

**Affiliations:** 1 Military Medical Support Center Neubrandenburg, Germany, Weg am Hang 35, Neubrandenburg, 17033, Germany, 49 395 372 3625; 2 Department of General Practice and Primary Care, University Medical Center Hamburg-Eppendorf, Hamburg, Germany; 3 Institute of General Practice, Universitätsmedizin Rostock, Rostock, Germany

**Keywords:** psychosocial risk factors, long-term illnesses, sick leave, general practice, return to work

## Abstract

**Background:**

Psychosocial risk factors increase the risk of long-term illnesses and prolonged return-to-work processes, with serious consequences for individuals as well as society. In general practice, psychosocial risk factors are captured comprehensively because general practitioners usually care for patients for years and know the patients’ entire social environment. Numerous studies have been conducted on aspects of psychosocial risk factors. However, a comprehensive, unrestricted summary of psychosocial risk factors is currently lacking.

**Objective:**

The present review aims to address this gap by providing an overview of psychosocial risk factors with relevance for Western and Western-oriented countries.

**Methods:**

This scoping review will be conducted in accordance with the methodology outlined in the Joanna Briggs Institute *Manual for Evidence Synthesis* in a 3-stage search strategy. Searched databases were MEDLINE, the Cochrane Library, Embase, and Web of Science. Each reference will be screened by 2 reviewers independently. The findings will be reported according to the PRISMA-ScR (Preferred Reporting Items for Systematic Reviews and Meta-Analyses extension for Scoping Reviews) guidelines.

**Results:**

A literature search conducted on September 8, 2025, resulted in 8515 articles after deduplication. Title and abstract screening started on September 10, 2025. It is estimated that screening will be completed in April 2026, followed by 2 to 3 months to complete data extraction and 1 to 2 months to draft the manuscript. The goal is to submit the scoping review manuscript in September 2026. We expect to identify various psychosocial aspects affecting long-term illnesses and return-to-work processes.

**Conclusions:**

Psychosocial risk factors play an important role in the development of long-term illnesses and prolonged return-to-work processes as they are often not communicated by patients but may influence the severity, duration, and progression of long-term illnesses. Evidence-based knowledge about comprehensive psychosocial risk factors will increase awareness of psychosocial aspects among physicians treating patients with long-term illnesses. This will help identify multidisciplinary prevention strategies for patients with long-term illnesses and relieve return-to-work processes.

## Introduction

### Background

The influence of psychosocial risk factors on the chronification of illnesses, the number of sick days taken, and return-to-work processes is widely accepted in contemporary medicine. Understanding of the genesis of illness as a dynamic interaction among biological, psychological, and social factors was shaped by the “biopsychosocial model of illness and health” established by Engel [1] in 1977. Even though the biopsychosocial model was primarily developed as a guiding principle of psychiatry, enormous efforts have been made over the past 20 years to create a biopsychosocial understanding in human medicine. The wide acceptance of the importance of the psychological and social dimensions of illnesses is reflected in the approval of the International Classification of Functioning, Disability, and Health (ICF) by all World Health Organization (WHO) member states in 2001 [2] and the embedding of the biopsychosocial model and the ICF in national laws, such as in Australia in the National Disability Insurance Scheme Act [3] and in Germany in the Social Code IX (amendment act: Federal Participation Act) [4]. Due to European Union Directive 89/391/EEC and its implementation in national occupational health and safety laws (eg, the Swedish Work Environment Act, the German Occupational Safety and Health Act, and Belgium’s Code on Well-Being at Work), comprehensive data focusing on psychological workplace-related risk factors [5] are currently available for European countries with a geographical concentration in Scandinavia. In recent years, further research focuses have been set on psychosocial disease components of chronic pain disorders such as chronic lower back pain and cardiovascular diseases [6,7].

To ensure that the terminology used is understood consistently, some definitions need to be clarified. In human medicine, risk factors are defined as precursors and predictors of disease. According to Kraemer et al [8], the most common definition of a “risk factor” refers to a factor that must be present before the onset of the disease and has to increase the probability of developing a disease. The psychosocial consequences of diseases, such as psychosocial distress caused by cancer, must be distinguished from this. While there is a notable overlap between the terms “chronic disease” and “long-term illness,” it is important to note that they cannot be equated. The term “chronic disease” describes noncommunicable diseases of long duration [9] without requiring any reference to ability or inability to work, whereas long-term illness is indirectly defined by the end of continued wage payments by the employer and the start of cost coverage by health insurance funds. Owing to the variability of legal requirements, there is no internationally valid definition of long-term illness. In the United States and Canada, there are no federal legal requirements for so-called sick pay, whereas there are established sick leave policies for all European countries, but the start of cost coverage by health insurance funds varies greatly. In Bulgaria, for example, sick pay is covered by the National Social Security Institute from the third day of absence. In Germany, health insurance funds pay for sick leave for more than 42 days, whereas in Luxembourg, sick pay is paid by the National Health Fund from the 78th day of illness [10]. According to the definition of long-term sick leave, data and research on this subject exist particularly for countries with an established sick pay system and absence records. Countries recording sick leave days report an increasing number of sick leave days over the recent years [11], with an expected new maximum for the year 2025. An examination of the distributions of sick days and sick cases revealed that a large share of sick leave days is caused by only a small number of cases: the long-term sick leave cases. For example, the largest health insurance fund in Germany reported that long-term illnesses (defined as >42 days of sick leave) were responsible for 3.5% of sick leave cases in 2024 but for 44.8% of sick leave days in 2024 [12]. This does not seem to be only an issue of an aging population. Even for young populations, for example, for a study population of soldiers of the German armed forces (average age of 28.57 years), a similar ratio was reported, with 2.67% of sick leave cases causing 25.11% of sick leave days by patients with long-term illnesses [13]. Long-term illnesses have a significant effect on the health care system and manifold effects on society as a whole (eg, through production downtime costs, as well as on patients themselves). In addition to the loss of participation in working life, effects on family [14], social activities, education, salary, and promotions [15] have been demonstrated. Participation in working life is important for economic independence and social contact. In particular, patients with mental illnesses seem to benefit from employment and reemployment [16]. With an early and successful return-to-work process, the duration of sick leave and, thereby, the negative consequences of long-term sick leave can be reduced [17]. However, the success of a return-to-work process depends on multiple aspects, including the main diagnosis, as well as the applied variable measures and activities and the involved stakeholders, therapists, and physicians. General practitioners are usually responsible for the care of patients with long-term illnesses and accompany them before, through, and after sick leave and during return-to-work processes. It is a part of their assignment to detect psychosocial risk factors at an early stage of illness. However, research findings show that psychosocial risk factors are correctly identified in only one-fifth to one-half of patients by general practitioners [18]. Especially if psychological aspects are present, patients are still afraid of stigmatization and are reluctant to report them [19]. This and a lack of awareness of the psychosocial dimension of their condition can lead patients to conceal psychosocial risk factors when they are consulting their general practitioner. With patients’ nonreporting and a delayed consideration of psychosocial aspects by physicians, the risk of long-term sick leave and a prolonged return-to-work process can increase [20]. Overall, previous research has shown that, despite all efforts, return-to-work measures often fail. A study published in 2025 evaluated a multidisciplinary rehabilitation program for patients with chronic low back pain and reported that 50% of the patients returned to work [21]. Other studies, such as that by Linge et al [22], have attempted to engage new technologies such as virtual reality gaming as a potential method to influence return to work for individuals on long-term sick leave, but return-to-work processes continue to achieve relatively modest success, and the long-term effects of return-to-work processes remain uncertain. Berglund et al [23] reported a long-term effect for the multidisciplinary treatment of patients with mental disorders and/or chronic pain. However, a systematic review from 2025 could not confirm long-lasting effects for interventions in workers with mental illnesses concerning sick leave duration [24]. Due to the unsatisfactory results of return-to-work processes, a causal research approach using risk factor research for primary prevention has been established, with different challenges. Current research on risk factors and risk constellations, such as that by Epple et al [25], often concentrates on nonvariable risk factors such as age and sex and has comprehensive relevance for the diagnoses of mental, behavioral, and musculoskeletal disorders with a high-risk of leading to long-term illness. In addition to risk factors such as age or guideline-compliant primary diagnoses, which are comparatively easy to identify, the detection of psychosocial risk factors seems to be more difficult. To support physicians in detecting psychosocial risk factors, a scoping review from 2023 analyzed 22 validated assessments for identifying patients with psychosocial problems in general practice without a final recommendation on the use of a specific assessment [26]. This was, among other things, due to considerable heterogeneity in the definition of psychosocial problems. This causal research approach can also be expanded by research on protective factors for sickness absence, as done in another previously published systematic review focusing on work-related factors [27].

Comprehensive reviews about psychosocial risk factors to address the research question (which psychosocial risk factors influence long-term illnesses in Western and Western-oriented countries?) are currently not available.

### Objectives

This scoping review aims to provide a comprehensive overview of psychosocial risk factors that influence long-term illnesses and return-to-work processes in Western and Western-oriented countries in general practice. Despite the reference to the work environment, not only workplace-related risk factors but also psychological and social risk factors at the interpersonal, intrapersonal, and sociocultural levels should be recorded regardless of the underlying main diagnosis.

The overarching aim of the review is to increase the awareness of psychosocial risk factors and encourage practitioners to consciously address risk factors in the treatment of patients with long-term illnesses. Identifying psychosocial aspects can enable their consideration in prevention, therapy, and rehabilitation. This might reduce long-term illnesses and increase the effectiveness of return-to-work measures, resulting in positive, relieving effects for society; the health care system; and, above all, the patients themselves.

## Methods

### Study Design

We planned a scoping review addressing the research question above by applying the updated methodological guidelines of the Joanna Briggs Institute (JBI) Manual for Evidence Synthesis [28] and adapted elements of the following PRISMA (Preferred Reporting Items for Systematic Reviews and Meta-Analyses) 2020 extensions: PRISMA for Abstracts [29], PRISMA-P (Preferred Reporting Items for Systematic Reviews and Meta-Analyses Protocols) (Checklist 1) [30,31], and PRISMA-ScR (Preferred Reporting Items for Systematic Reviews and Meta-Analyses extension for Scoping Reviews) [32].

### Eligibility Criteria

The eligibility criteria were defined according to the JBI’s population, concept, and context framework. Papers fulfilling the following criteria will be considered.

#### Population

The population will be people with long-term illnesses. Owing to the inconsistent definition of long-term illness, all adult patients who were on sick leave or are on sick leave at the time of examination will be eligible for inclusion regardless of occupation, main diagnosis, current work status, and duration of sick leave. Consequently, the included population will be of working age (15-64 years) as defined by the Organisation for Economic Co-operation and Development [33].

#### Concept

The concept will be psychosocial risk factor. All types of psychosocial aspects, also using the terms “psychosocial problems,” “factors,” “risk factors,” and “complaints,” will be eligible for inclusion. Whether they meet the definition of a (psychosocial) risk factor will be assessed during the review process.

#### Context

The context will be general practice. This scoping review will encompass general practices and all comparable facilities (eg, family medicine and primary care) where patients receive treatment from physicians throughout the course of their disease that is not specialized in a single aspect of the long-term illness.

#### Study Characteristics

Research that examines populations in countries that are either Western or Western oriented (North Atlantic Treaty Organization countries, as well as Australia, Austria, Ireland, Liechtenstein, New Zealand, and Switzerland) will be included due to the comparability of societal structures, safety nets and family structures, work environments, and scientific approaches as a result of standardized questionnaires mostly developed in Western countries. Furthermore, only research published after January 1, 2005, will be included to identify psychosocial risk factors with high relevance for contemporary practice. There will be no search restrictions with respect to study design or language. According to these inclusion criteria, the scoping review will exclude studies with a focus on (chronic) illnesses without reference to (long-term) sick leave, as well as studies that focus solely on populations outside the working age range (ie, <15 years and >64 years) and patients, health care professionals and medical institutions in the settings of specialist or hospital care (eg. occupational physicians, emergency departments, tertiary care hospitals), studies not reporting psychosocial risk factors, studies focused on populations solely residing in non-Western or non-Western-oriented countries, and research published before January 2005.

### Information Sources

The following databases were searched: MEDLINE, the Cochrane Library (CENTRAL and CDSR), and Embase. In addition, the Web of Science Core Collection database was included to establish a comprehensive search. The searches of MEDLINE and Embase were conducted via Ovid. The Cochrane Library and Web of Science were searched via their own search engines. Database-specific syntaxes were used, and key search terms were used for each database, in addition to MeSH or Emtree terms. Ongoing studies were incorporated as registrations in ClinicalTrials.gov and the WHO register (International Clinical Trials Registry Platform [ICTRP]) via the Cochrane Library search. Gray literature, such as conference proceedings, was not considered in this review. A test search of all the databases was carried out on July 28, 2025. The final search was conducted on September 8, 2025. All databases were searched from January 1, 2005, to September 8, 2025.

### Search Strategy

As recommended by the JBI, a 3-stage search strategy was used.

After a preliminary search in MEDLINE using the terms “psychosocial” [Title/Abstract] AND “general practice” [MeSH Terms] AND “long-term disease” [Title/Abstract], the first 50 results (sorted by best match) were screened for text words in titles and abstracts, author keywords, and MeSH terms to identify appropriate search terms.

An experienced information specialist (JN) subsequently assisted in formulating a sensitive search strategy using the identified free-text terms and MeSH terms. On the basis of team discussions, additional controlled synonyms and similar terms were added according to the eligibility criteria to sensitize and specify the search string for MEDLINE. Furthermore, psychosocial factors not labeled as psychosocial risk factors were included by adding them not only with the umbrella term psychosocial risk factors but also as individual, specific factors identified as psychosocial risk factors. The latter included social isolation, mental stress, bullying, poverty, educational level, and workload. A pilot search was conducted in MEDLINE, followed by a validation of the search strategy via a sample of 8 eligible studies that were previously identified as relevant at the title and abstract screening stage. The resulting MEDLINE search string was manually translated by the information specialist for the Embase, Cochrane Library, and Web of Science databases and subsequently tested and adjusted across all these databases to increase the specificity of the results.

The final search strategy comprised the following elements:

- Population (long-term illnesses): “(long-term, synonyms for long-term) AND (illness, synonyms for illness) AND (work, synonyms for work) OR (chronic disease AND (work, synonyms for work)) OR (absenteeism, synonyms for absenteeism)”
- Concept (psychosocial risk factors): “((psych*, synonym for psychological) OR soci*) AND (risk factors, synonyms for risk factors) OR (psychosocial risk factors namely)”
- Context (general practice): “(general practice, synonyms for general practice) OR (family practice, synonyms for family practice) OR (primary care, synonyms for primary care)”

Multimedia Appendix 1 shows the final search string used for the MEDLINE database on the Ovid platform.

In the third stage, the reference lists of all included articles after full-text screening will be searched to identify articles that were previously missed in an ascendancy approach.

### Study Records

#### Data Management

The search results were exported from the databases and uploaded to the Citavi 6 citation and reference management tool (Swiss Academic Software GmbH). Duplicates were eliminated via the deduplication function of Citavi. After uploading the remaining references to Covidence (Veritas Health Innovation), its automatic duplicate detection function identified further duplicates, which were removed after a manual check. Covidence was also used to manage references during the selection and data collection processes.

#### Selection Process

Titles and abstracts will be independently screened by at least 2 reviewers to determine whether the studies meet the predetermined eligibility criteria. Reviewers will be blinded to each other’s decisions. After screening the first 300 results, the reviewers’ consensus will be evaluated. If there is less than 75% agreement, the eligibility criteria will be discussed and refined. If the eligibility criteria are refined, the agreement rate will be checked again after screening the next 300 articles until an acceptable agreement rate (ie, ≥75%) is achieved. Articles approved by both reviewers as “yes” or “maybe” will proceed directly to the full-text stage. Articles on which the reviewers disagree (1 ”yes” or ”maybe” and 1 “no”) will be resolved by an impartial reviewer acting as a tiebreaker. Admitted articles will undergo independent full-text reading to determine final inclusion in the scoping review. Any disagreements will be resolved through team discussion. If no agreement can be reached, the tiebreaker reviewer will be consulted. For each study excluded at the full-text stage, one reason for exclusion will be reported, together with the name of the first author and publication year in tabular format. The contents of the excluded studies will not be reported in the final review. The PRISMA flowchart will be used for the documentation of the selection process.

#### Data Collection Process

Data collection will be conducted via the Covidence data collection function. Two reviewers will pretest the data extraction form on a small sample of included literature to ensure that all relevant data are captured adequately. The remaining data collection process for the included papers will be carried out by one reviewer and checked by a second reviewer. Modifications to the data collection process are possible after team discussion and must be reported in the resulting scoping review.

### Data Items

Basic publication data (author, publication date, study start and end date, country, study design, setting, and number of participants), participant characteristics (age, gender, nationality, occupation, working status, and main diagnosis), investigated psychosocial risk factors, assessments, interventions, study aims, reported results, key conclusions, funding sources, name of extractor, and date of extraction will be documented supported by the Covidence data extraction tool. Any disagreements will be resolved through discussion or by involving the tiebreaker reviewer if agreement cannot be reached through discussion. In case of missing data, it is not planned to contact the authors to request missing or additional data.

### Outcomes and Prioritization

Primary outcomes include reported psychosocial risk factors and duration of associated sick leave. Secondary outcomes such as improvement in quality of life will also be reported.

### Risk of Bias in Individual Studies

Critical appraisal of individual studies is not planned owing to the scoping review aims.

### Data Synthesis

During the data synthesis process, all extracted data items will be categorized and analyzed via frequency counts. The identified psychosocial risk factors will be divided into the subcategories of interpersonal (eg, social isolation, family conflicts, and bullying), intrapersonal (eg, anxiety and fear avoidance belief), and sociocultural (eg, socioeconomic status and educational level) risk factors. The characteristics of the included studies will be displayed in tabular format. A descriptive, systematic summary will be provided to explain the characteristics and findings of the included studies.

## Results

The final search of all databases was conducted on September 8, 2025, and retrieved 14415 documents: 5188 references from Embase, 4673 records from MEDLINE, and 3573 records from Web of Science Core Collection. The Cochrane Library search identified 953 trials and 28 reviews. The trial records included 449 records identified for CENTRAL from PubMed, 319 records identified from Embase, 259 records identified from ClinicalTrials.gov, 128 records identified from the WHO study register (ICTRP), and 3 records from CINAHL. Following the removal of duplicates, the final search identified 8515 references for title and abstract screening, as shown in Table 1.

**Table 1.** Search results per database (N=14,415).

Database	Host	Date	Hits, n (%)
Embase	Ovid	September 8, 2025	5188 (35.99)
Medline	Ovid	September 8, 2025	4673 (32.42)
Web of Science Core Collection (Science Citation Index Expanded; Social Sciences Citation Index)	Clarivate	September 8, 2025	3573 (24.79)
Cochrane Library	Cochrane	September 8, 2025	953 trials (6.61); 28 reviews (0.19); 981 in total (6.81)
Total	—^a^	—	14415 (100)
After deduplication	—	—	8515 (59.07)

a Not applicable.

After screening the first 247 references, the calculated agreement rate was 77.33%, so the set goal (75%) was achieved. The agreement rate will be evaluated again during the screening process. Title and abstract screening started on September 10, 2025. It is estimated that screening will be completed in April 2026, followed by 2 to 3 months to complete data extraction and 1 to 2 months to draft the manuscript. The goal is to submit the scoping review manuscript in September 2026. No external funding was received for this scoping review protocol.

## Discussion

### Achievement of Objectives

The aim of this scoping review is to provide a broad overview of psychosocial risk factors that function as moderators of sick leave duration and return-to-work processes due to long-term illnesses in Western and Western-oriented countries. With a total of 14,415 references identified in the final search and 8515 remaining references after deduplication, the search results are in the upper range compared with other scoping reviews that examine psychosocial topics [34-36]. The comparably high number of included references indicates that the aim of capturing multiple psychosocial risk factors in a broad overview seems to be achievable.

### Comparison to Prior Work

Although there is comparability with other reviews about psychosocial risk factors regarding the quantitative search results, existing research is content-wise not comparable, especially based on two specific content features. To date, research has focused mostly on specific aspects of psychosocial risk factors (assessments [26], interventions [24], work-related aspects [27], and special main diagnoses [37]), and only a limited number of studies reference the work environment and sick leave. However, the results and conclusions of current publications require further research to enhance the detection of psychosocial risk factors and their inclusion in treatment. Recent studies evaluating the outcomes of rehabilitation measures have shown a decrease in the number of sick leave days [38] as well as an increase in relative weekly working hours [39], but the percentage of patients returning to work after rehabilitation has been reported as 37% to 85% [40]. The effectiveness of rehabilitation seems to depend on demographics; the type of injury or disease; the type of rehabilitation measures; the rehabilitation setting; and many other physical, psychological, and social factors [41]. The fact that rehabilitation measures are subjected to multiple influences is consistent with the understanding of functionality, disability, and health as a dynamic interaction process. However, a recent meta-analysis reported no statistically significant difference between multidisciplinary rehabilitation and usual care [42].

### Strengths and Methodological Challenges

Due to the moderate outcomes of rehabilitation measures for patients with long-term illnesses, we assumed that further evidence-based knowledge about psychosocial risk factors that influence long-term sick leave and return-to-work processes is required for the prevention, treatment, and rehabilitation of patients at risk of long-term sick leave. To generate this knowledge, a comprehensive search string was developed in an iterative and exploratory process with the aim of capturing all psychosocial risk factors with high relevance for the population and setting of interest. This led to different challenges, which were resolved as described below. All three population, concept, and context elements were complex in the development of the search strategy because the key terms (“long-term illness,” “general practice,” and “psychosocial risk factor”) combine different meanings, which can vary between different countries and health care systems. Evaluating the search string quality after the primary test search showed a low specificity for the population element. Many results were generated from the terms “chronic disease” and other synonyms for long-term illness, with a low reference to the work environment. To establish a connection between the population of people with long-term illnesses and the world of work, the terms for long-term illnesses were restricted to the context of “work” and “employment.” By adding the terms “absenteeism,” “sick leave,” and “return-to-work,” several references considered relevant for this scoping review were found. To depict the context comprehensively, we used different terms for “general practice” and “family practice” and added the system of primary health care because many countries relevant to this scoping review established primary health care according to the recommendation of the United Nations General Assembly resolution, which designates primary health care as “the most inclusive, effective and efficient approach to enhance people’s physical and mental health...” [43]. The setting of general practice was selected because general practitioners often know patients’ medical history for generations and patients’ psychosocial background. We expect this to enable us to generate a comprehensive overview of psychosocial risk factors with relevance not only for general practitioners but also for other practitioners specialized in the various levels of prevention. However, the most challenging element of this review was the concept of psychosocial risk factors. Because psychosocial risk factors are not always labeled as psychosocial risk factors, many of these had to be added namely, resulting in 2697 additional hits. The risk of generating many irrelevant hits for title and abstract screening by enlarging the search string with specific psychosocial risk factors was accepted by the team to reach high sensitivity for different psychosocial risk factors. Analyzing the search results after adding the psychosocial risk factors namely revealed that “mental diseases” and “depression” generated many hits focused on psychiatric issues (antidepressant treatment and dementia) without examining these topics as risk factors; therefore, these two terms were included in the search string by restricting them as comorbidities.

### Limitations

Consistent with the methodology for scoping reviews, no formal quality appraisal will be conducted, meaning that the scoping review will map psychosocial risk factors without formal assessment of the methodological quality or risk of bias of the included studies. The heterogeneity and imprecise definitions of psychosocial risk factors will be a major challenge in examining them throughout the screening process. Owing to the mostly retrospective examinations after the onset of an illness, the chronological requirement according to the definition of Kraemer et al [8], which requires the existence of the risk factor before the onset of an illness, is often not verifiable [44]. This will result in a partially subjective rating of a psychosocial risk factor. Furthermore, the enormous research field of psychosocial risk factors and their incongruent wording carries the risk of failing to account for all of them. The expected high number of different risk factors will also make categorization necessary, which can result in a loss of information because of simplified reporting. Other expected difficulties include low objectivity (resulting from examinations, mostly via self-assessment, often without validation of assessments [26,34]) and interactions between psychosocial risk factors and long-term illnesses (eg, social isolation may prolong sick leave, but long-term sick leave may increase social isolation). Proving causality is difficult in classic risk factor research because the implementation of randomized controlled studies is methodologically not possible due to ethical violations, long latency periods, and practical feasibility and compliance. As a specification, we included references published in the last 20 years (registry on the Open Science Framework in the past 30 years) according to the test search screening results for all databases and the team members’ experience to capture especially psychosocial risk factors with high relevance for today’s issue.

Nevertheless, we assume that this scoping review will be highly sensitive as a large number of studies were identified, including all relevant references known to the review team, and as the search string was developed in collaboration with an expert based on general rules and recommendations [45]. The precision of the search string will be calculated after screening is completed.

### Future Directions and Dissemination

Despite existing knowledge about psychosocial risk factors, further expanding and structured knowledge about potential psychosocial risk factors may increase awareness among (general) practitioners and motivate them to influence psychosocial risk factors at best in the sense of primary prevention. Research shows that even addressing psychosocial aspects in general practice increases patient satisfaction [46] and leads to greater patient motivation for change, a better understanding of complex relationships between symptoms and causes, more open communication with the general practitioner, and higher therapy adherence [47]. However, addressing psychosocial risk factors often creates just a short-term effect. To achieve a long-term effect, further interventions are necessary. This finding also indicates that knowledge about causal psychosocial risk factors can serve as the basis for further research, including improvements in intervention and rehabilitation studies and in the implementation of structured follow-up care of patients after a successful return to work. The assumption that a comprehensive collection, listing, and categorization of psychosocial risk factors can influence return to work should be substantiated by further evaluations specifically on return-to-work processes. As the avoidance and reduction of long-term illnesses have various effects, such as a reduction in health care system load and in the consequences of demographic change due to an aging workforce and a shortage of skilled workers, as well as the overall ability of the patient to exercise their right to participate and socially integrate, measures to avoid, reduce, or shorten long-term illnesses should be further addressed with respect to psychosocial risk factors. In the future, psychosocial risk factors will continue to play an important role, perhaps in new contexts, as evidenced in the context of the COVID-19 pandemic. The pandemic and the lockdowns massively increased the prevalence of specific psychosocial risk factors, such as social isolation, and diseases such as the postacute sequelae of COVID-19 are difficult to explain without the influence of psychosocial risk factors [48].

### Conclusions

A systematic examination of the present research question to provide comprehensive knowledge of psychosocial risk factors for long-term illnesses might reduce the latter by elevating the awareness of practitioners directly, but could also constitute the basis for future research.

Any changes to this review protocol will be reported in the final scoping review.

## Supplementary material

10.2196/102585 Multimedia Appendix 1 Search string for MEDLINE (Ovid).

10.2196/102585 Checklist 1 PRISMA-P checklist.

## Abbreviations

ICF: International Classification of Functioning, Disability, and Health
ICTRP: International Clinical Trials Registry Platform
JBI: Joanna Briggs Institute
PRISMA: Preferred Reporting Items for Systematic Reviews and Meta-Analyses
PRISMA-P: Preferred Reporting Items for Systematic Reviews and Meta-Analyses Protocols
PRISMA-ScR: Preferred Reporting Items for Systematic Reviews and Meta-Analyses extension for Scoping Reviews
WHO: World Health Organization
