# Supplementary material for: The Impact of Psychosocial Aspects as Risk Factors for Long-Term Illnesses in General Practice: Protocol for a Scoping Review
Source: JMIR Res Protoc. 2026 Oct 21;15:e102585. doi: 10.2196/102585 (PMC13686610; doi:10.2196/102585)
Supplement: Supplementary file 1 [file resprot-v15-e102585-s001.pdf]

**Supporting information S1 Table: Search string Medline (Ovid)**

Ovid MEDLINE(R) ALL <1946 to September 05, 2025>

| Search | Search terms | Items found |
|---|---|---|
| 1 | (longterm or long-term).ti,ab,kf. | 1147781 |
| 2 | extended.ti,ab,kf. | 369643 |
| 3 | prolonged.ti,ab,kf. | 404545 |
| 4 | protracted.ti,ab,kf. | 18276 |
| 5 | chronic.ti,ab,kf. | 1582008 |
| 6 | or/1-5 | 3285192 |
| 7 | illness*.ti,ab,kf. | 364466 |
| 8 | diseas*.ti,ab,kf. | 5536564 |
| 9 | disorder*.ti,ab,kf. | 1669961 |
| 10 | health problem.ti,ab,kf. | 70058 |
| 11 | complain*.ti,ab,kf. | 164651 |
| 12 | or/7-11 | 7029518 |
| 13 | 6 and 12 | 1271788 |
| 14 | exp work/ | 74236 |
| 15 | exp employment/ | 107197 |
| 16 | 14 or 15 | 170544 |
| 17 | (workrelated or work-related).ti,ab,kf. | 24937 |
| 18 | employ*.ti,ab,kf. | 975232 |
| 19 | 17 or 18 | 993431 |
| 20 | 13 and (16 or 19) | 38983 |
| 21 | exp chronic disease/ | 669023 |
| 22 | 21 and 16 | 2373 |
| 23 | exp absenteeism/ | 10181 |
| 24 | exp sick leave/ | 7241 |
| 25 | exp return to work/ | 4260 |
| 26 | absen*.ti,ab,kf. | 933106 |
| 27 | sick*.ti,ab,kf. | 105284 |
| 28 | (return adj3 work).ti,ab,kf. | 13572 |
| 29 | or/23-28 | 1048129 |
| 30 | 20 or 22 or 29 | 1084953 |
| 31 | exp general practice/ | 80588 |
| 32 | exp general practitioners/ | 12093 |
| 33 | general pract*.ti,ab,kf. | 100793 |
| 34 | general physician*.ti,ab,kf. | 2242 |
| 35 | exp family practice/ | 67993 |
| 36 | exp physicians, family/ | 17645 |
| 37 | family pract*.ti,ab,kf. | 11150 |
| 38 | family physician*.ti,ab,kf. | 17776 |
| 39 | family med*.ti,ab,kf. | 17753 |
| 40 | exp primary health care/ | 208544 |
| 41 | primary care.ti,ab,kf. | 165833 |
| 42 | primary health care.ti,ab,kf. | 38666 |
| 43 | or/31-42 | 456490 |
| 44 | psych*.ti,ab,kf. | 1186065 |
| 45 | mental*.ti,ab,kf. | 585141 |
| 46 | soci*.ti,ab,kf. | 1535532 |
| 47 | or/44-46 | 2754369 |
| 48 | exp risk factors/ | 1053663 |
| 49 | exp comorbidity/ | 135671 |
| 50 | factor*.ti,ab,kf. | 4819940 |
| 51 | aspect*.ti,ab,kf. | 776133 |
| 52 | problem*.ti,ab,kf. | 1366882 |
| 53 | complain*.ti,ab,kf. | 164651 |
| 54 | comorb*.ti,ab,kf. | 285102 |

| | | |
|---|---|---|
| 55 | or/48-54 | 7347343 |
| 56 | 47 and 55 | 1081717 |
| 57 | exp socioeconomic factors/ | 540351 |
| 58 | exp mental disorders/ | 1564891 |
| 59 | exp comorbidity/ | 135671 |
| 60 | 58 and 59 | 46355 |
| 61 | exp depression/ | 173364 |
| 62 | 59 and 61 | 7233 |
| 63 | exp fear/ | 43455 |
| 64 | exp anxiety/ | 129570 |
| 65 | exp anxiety disorders/ | 96906 |
| 66 | exp stress, psychological/ | 168660 |
| 67 | exp sleep wake disorders/ | 121338 |
| 68 | exp smoking/ | 167693 |
| 69 | exp alcohol drinking/ | 82773 |
| 70 | exp "feeding and eating disorders"/ | 39576 |
| 71 | exp life style/ | 120284 |
| 72 | exp conflict, psychological/ | 17207 |
| 73 | exp parenting/ | 23781 |
| 74 | exp social isolation/ | 28861 |
| 75 | exp social environment/ | 136100 |
| 76 | exp racism/ | 8637 |
| 77 | exp social problems/ | 377078 |
| 78 | exp "personnel staffing and scheduling"/ | 48823 |
| 79 | exp social discrimination/ | 16248 |
| 80 | exp job satisfaction/ | 31121 |
| 81 | exp "dissent and disputes"/ | 9509 |
| 82 | or/63-81 | 1406058 |
| 83 | fear.ti,ab,kf. | 100852 |
| 84 | anxiety.ti,ab,kf. | 319582 |
| 85 | psychological stress.ti,ab,kf. | 12502 |
| 86 | sleep wake disorder*.ti,ab,kf. | 821 |
| 87 | smoking.ti,ab,kf. | 296122 |
| 88 | alcohol drinking.ti,ab,kf. | 9296 |
| 89 | eating disorder*.ti,ab,kf. | 30367 |
| 90 | (lifestyle* or life-style*).ti,ab,kf. | 179008 |
| 91 | conflict*.ti,ab,kf. | 177092 |
| 92 | parenting.ti,ab,kf. | 31665 |
| 93 | social isolation.ti,ab,kf. | 16274 |
| 94 | social environment.ti,ab,kf. | 9798 |
| 95 | racism.ti,ab,kf. | 12138 |
| 96 | ((personnel or staff*) adj4 schedul*).ti,ab,kf. | 1129 |
| 97 | social discrimination.ti,ab,kf. | 1315 |
| 98 | job satisfaction.ti,ab,kf. | 14157 |
| 99 | social support.ti,ab,kf. | 65667 |
| 100 | social communication.ti,ab,kf. | 5578 |
| 101 | social class.ti,ab,kf. | 11059 |
| 102 | addict*.ti,ab,kf. | 91810 |
| 103 | abuse.ti,ab,kf. | 154480 |
| 104 | divorce.ti,ab,kf. | 6241 |
| 105 | partnership.ti,ab,kf. | 36086 |
| 106 | discriminat*.ti,ab,kf. | 335151 |
| 107 | bully*.ti,ab,kf. | 10305 |
| 108 | viol*.ti,ab,kf. | 157504 |
| 109 | financ*.ti,ab,kf. | 173058 |
| 110 | poverty.ti,ab,kf. | 40023 |
| 111 | income.ti,ab,kf. | 218678 |
| 112 | economic status.ti,ab,kf. | 19988 |
| 113 | debts.ti,ab,kf. | 695 |

| | | |
|---|---|---|
| 114 | at work.ti,ab,kf. | 25008 |
| 115 | workplace*.ti,ab,kf. | 65779 |
| 116 | working condition*.ti,ab,kf. | 16334 |
| 117 | work environment.ti,ab,kf. | 14139 |
| 118 | (workload or work-load).ti,ab,kf. | 45013 |
| 119 | (worklife or work-life).ti,ab,kf. | 6311 |
| 120 | working hours.ti,ab,kf. | 7925 |
| 121 | educ* level.ti,ab,kf. | 50652 |
| 122 | educ* attainment.ti,ab,kf. | 14940 |
| 123 | or/83-122 | 2317463 |
| 124 | or/56-57,60,62,82,123 | 3856995 |
| 125 | 30 and 43 and 124 | 6124 |
| 126 | limit 125 to dt=20050101-20250908 | 4673 |
