# Supplementary material for: The Impact of Psychosocial Aspects as Risk Factors for Long-Term Illnesses in General Practice: Protocol for a Scoping Review
Source: JMIR Res Protoc. 2026 Oct 21;15:e102585. doi: 10.2196/102585 (PMC13686610; doi:10.2196/102585)
Supplement: Supplementary file 2 [file resprot-v15-e102585-s002.pdf]

PRISMA-P (Preferred Reporting Items for Systematic review and Meta-Analysis Protocols) 2015 checklist: recommended items to address in a systematic review protocol*

Section and topic	Item No	Checklist item	Page number
**ADMINISTRATIVE INFORMATION**			
Title:		**Full title: The impact of psychosocial aspects as risk factors for long-term illnesses in general practice: A scoping review protocol**<br>**Short title: Psychosocial risk factors for long-term illnesses in general practice: A scoping review protocol**	
Identification	1a	Identify the report as a protocol of a scoping review: **Yes**	1
Update	1b	If the protocol is for an update of a previous systematic review, identify as such: **Not applicable**	-
Registration	2	If registered, provide the name of the registry (such as PROSPERO) and registration number<br>**In accordance with the guidelines, this scoping review protocol was registered with the Open Science Framework (OSF) 2025 June 27: https://doi.org/10.17605/OSF.IO/FSYM6**	3
Authors:		**Kathrin Epple, Julia Nothacker, Christin Löffler, Lisa Richter, Ralf Jendyk, Andreas Dierich**	1
Contact	3a	Provide name, institutional affiliation, e-mail address of all protocol authors; provide physical mailing address of corresponding author:<br>**Kathrin Epple[1] kathrinepple@bundeswehr.org**<br>**Julia Nothacker[2], j.nothacker@uke.de**<br>**Christin Löffler[3], christin.loeffler@med.uni-rostock.de**<br>**Lisa Richter[3], lisa.richter@med.uni-rostock.de**<br>**Ralf Jendyk[3], ralf.jendyk@med.uni-rostock.de**<br>**Andreas Dierich[1] andreasdierich@bundeswehr.org**<br><br>**[1] Military Medical Support Center Neubrandenburg, Neubrandenburg, Germany**<br>**[2] Department of General Practice and Primary Care, University Medical Center Hamburg-Eppendorf, Hamburg, Germany**<br>**[3] Institute of General Practice, Rostock University Medical Center, Rostock, Germany**<br><br>*** Corresponding author**<br>**E-mail: kathrinepple@bundeswehr.org (KE)**	
Contributions	3b	Describe contributions of protocol authors and identify the guarantor of the review:<br>**AD: conceptualization, tie braker role and supervision of the review process**<br>**KE: preliminary research topic analyses, documentation and data curation, project administration, writing-original draft preparation**<br>**JN: search string development**	1

		**KE, JN, CL, LR, RJ, AD: final conception of the review, screening, reviewing, writing-review and editing**	
Amendments	4	If the protocol represents an amendment of a previously completed or published protocol, identify as such and list changes; otherwise, state plan for documenting important protocol amendments: **Not applicable**	-
Support:		**Not applicable**	-
Sources	5a	Indicate sources of financial or other support for the review	
Sponsor	5b	Provide name for the review funder and/or sponsor	
Role of sponsor or funder	5c	Describe roles of funder(s), sponsor(s), and/or institution(s), if any, in developing the protocol	
**INTRODUCTION**			
Rationale	6	Describe the rationale for the review in the context of what is already known<br>**Long-term illnesses are often prolonged by the presence of psychosocial risk factors with serious consequences for the patients and society. Numerous studies have been conducted on specific aspects of psychosocial risk factors. however, a comprehensive summary of psychosocial risk factors in the setting of general practice is currently lacking.**	4
Objectives	7	Provide an explicit statement of the question(s) the review will address with reference to participants, interventions, comparators, and outcomes (PICO)<br>**The review aims to provide a comprehensive, unrestricted overview of psychosocial risk factors (C) with relevance for long-term illnesses (P) in general practices (C)**	7
**METHODS**			
Eligibility criteria	8	Specify the study characteristics (such as PICO, study design, setting, time frame) and report characteristics (such as years considered, language, publication status) to be used as criteria for eligibility for the review<br>**Study characteristics:**<br>**Population: Patients with long-term illnesses on sick-leave**<br>**Concept: psychosocial risk factors**<br>**Context: general practice and primary care**<br>**Study design: Scoping review**<br>**Time Frame: Preparations started March 2025, Search was conducted September 2025, Screening started October 2025 and is planned to be completed April 2026, Extraction and writing is planned to be finished at the end of 2026**<br><br>**Report characteristics of included publications:**<br>**Research published after January 1, 2005**<br>**Research examining populations in countries that are either Western or Western-oriented countries (North Atlantic Treaty Organization (NATO) countries, as well as Australia, Austria, Ireland, Liechtenstein, New Zealand and Switzerland)**<br>**No search restrictions with respect to study design, publication status or language**	8

Information sources	9	Describe all intended information sources (such as electronic databases, contact with study authors, trial registers or other grey literature sources) with planned dates of coverage<br>**Medline (Ovid), the Cochrane Library (the Cochrane Central Register of Controlled Trials (CENTRAL) and the Cochrane Database of Systematic Reviews (CDSR)), Embase (Ovid), the Web of Science Core Collection**<br>**All databases were searched from January 1, 2005 to September 8, 2025**<br>**Contact to the authors to request missing or additional data: not planned**<br>**Trial registers: registrations considered in clinicaltrials.gov and the WHO register (International Clinical Trials Registry Platform (ICTRP)) via the Cochrane Library search**<br>**Gray literature (such as conference proceedings): not considered**	8
Search strategy	10	Present draft of search strategy to be used for at least one electronic database, including planned limits, such that it could be repeated: **please check supporting information S1 for the final search string used for the Medline database (Ovid)**	8
Study records:			
Data management	11a	Describe the mechanism(s) that will be used to manage records and data throughout the review<br>**Literature management: Citavi 6**<br>**Deduplication: Citavi 6 and Covidence**<br>**Selection and data collection: Covidence**	11
Selection process	11b	State the process that will be used for selecting studies (such as two independent reviewers) through each phase of the review (that is, screening, eligibility and inclusion in meta-analysis)<br>**Title/Abstract and full text screening by two independent reviewers, blinded to each other´s decision**<br>**Disagreements at Title/Abstract screening: resolved by a third reviewer with a tie braker function**<br>**Disagreements at full-text stage: Team discussion, if no agreement can be reached, tie breaker reviewer will be consulted**<br>**The PRISMA flow chart will be used for the documentation of the selection process.**	11
Data collection process	11c	Describe planned method of extracting data from reports (such as piloting forms, done independently, in duplicate), any processes for obtaining and confirming data from investigators<br>**Data collection: Covidence data collection function, pretested by two reviewers, continued by one reviewer, checked by a second reviewer**	11
Data items	12	List and define all variables for which data will be sought (such as PICO items, funding sources), any pre-planned data assumptions and simplifications<br>**Basic publication data (author, publication date, study start and end date, country, study design, setting, number of participants), participant characteristics (age, gender, nationality, occupation, working status, main diagnosis), investigated psychosocial risk factors, assessments, interventions, study aims, reported results, key conclusions, funding sources, name of extractor and date of extraction**	12
Outcomes and prioritization	13	List and define all outcomes for which data will be sought, including prioritization of main and additional outcomes, with rationale<br>**Primary outcomes: reported psychosocial risk factors and duration of associated sick leave (according to the objective of the scoping review)**	12

		**Secondary outcomes: improvement in quality of life (to achieve the aim of a comprehensive overview about evidence-based knowledge about psychosocial risk factors)**	
Risk of bias in individual studies	14	Describe anticipated methods for assessing risk of bias of individual studies, including whether this will be done at the outcome or study level, or both; state how this information will be used in data synthesis: **Not applicable**	-
Data synthesis	15a	Describe criteria under which study data will be quantitatively synthesised: **Not applicable**	-
	15b	If data are appropriate for quantitative synthesis, describe planned summary measures, methods of handling data and methods of combining data from studies, including any planned exploration of consistency (such as I^2, Kendall's τ)	
	15c	Describe any proposed additional analyses (such as sensitivity or subgroup analyses, meta-regression)	
	15d	If quantitative synthesis is not appropriate, describe the type of summary planned<br>**Extracted data items (psychosocial risk factors) will be analyzed via frequency counts and categorized into the subcategories of interpersonal (for example, social isolation, family conflicts, bullying), intrapersonal (for example, anxiety, fear-avoidance belief) and sociocultural (for example, socioeconomic status, education level) risk factors**<br>**The characteristics of the included studies will be displayed in tabular format. A descriptive, systematic summary will be provided to summarize and explain the characteristics and findings of the included studies.**	12
Meta-bias(es)	16	Specify any planned assessment of meta-bias(es) (such as publication bias across studies, selective reporting within studies): **Not applicable**	-
Confidence in cumulative evidence	17	Describe how the strength of the body of evidence will be assessed (such as GRADE): **Not applicable**	-

*** It is strongly recommended that this checklist be read in conjunction with the PRISMA-P Explanation and Elaboration (cite when available) for important clarification on the items. Amendments to a review protocol should be tracked and dated. The copyright for PRISMA-P (including checklist) is held by the PRISMA-P Group and is distributed under a Creative Commons Attribution Licence 4.0.**

From: Shamseer L, Moher D, Clarke M, Ghersi D, Liberati A, Petticrew M, Shekelle P, Stewart L, PRISMA-P Group. Preferred reporting items for systematic review and meta-analysis protocols (PRISMA-P) 2015: elaboration and explanation. BMJ. 2015 Jan 2;349(jan02 1):g7647.
